# Cardio pulmonary resuscitation (CPR) in the frail and those with multiple health conditions: Outcomes before and during the COVID pandemic

**DOI:** 10.1016/j.clinme.2023.100001

**Published:** 2024-01-19

**Authors:** Aled Lloyd, Elin Thomas, Julia Scaife, Nicky Leopold

**Affiliations:** a Morriston Hospital, Swansea Bay University Health Board, Swansea, UK; b Singleton Hospital, Swansea Bay University Health Board, Swansea, UK

**Keywords:** Cardiopulmonary resuscitation, CPR, Frailty, Comorbid, Coronavirus

## Abstract

Coronavirus 2019 (COVID-19)-era resuscitation guidelines advised personal protective equipment before chest compressions and proactive advanced care planning. We investigated the impact of COVID-19 on cardiopulmonary resuscitation (CPR) outcomes according to scoring of frailty and of multiple health conditions. A retrospective single-centre analysis of clinical and electronic records for all adult cardiac arrest calls on wards between June 2020 and June 2021 was performed. Data were compared with a cohort pre-COVID (March 2017–March 2018). In total, 62 patients received CPR in 2020–21 compared with 113 in 2017–18. Similar rates of return of spontaneous circulation (ROSC) and a statistically insignificant survival increase from 23.8% to 32.2% (p=0.210). There were linear relationships between Clinical Frailty Scale (CFS) or Charlson Comorbidity Index (CCI) and diminished survival in the pooled data (both p<0.001). Both increasing frailty (measured by CFS) and comorbidity (measured by CCI) were associated with reduced survival from CPR. However, survival and ROSC during COVID-19 were no worse than before the pandemic.

## Introduction

Concerns about limited hospital resources, particularly critical care, meant that proactive discussion regarding resuscitation and ceiling of care discussions became increasingly important during the Coronavirus 2019 (COVID-19) pandemic. Advanced care planning in inpatients who are frail and/or who have multiple health conditions has been particularly pertinent, and significant research in this field has been undertaken. Over the past 4 years, there have been at least seven single-centre retrospective analyses comparing survival after cardiopulmonary resuscitation (CPR) among patients who are frail and those who are not.^1^, ^2^, ^3^, ^4^, ^5^, ^6^, ^7^ Although frailty as a term can be difficult to precisely define, Rockwood famously defined it as ‘a term widely used to denote a multidimensional syndrome of loss of reserves (energy, physical ability, cognition, health) that gives rise to vulnerability’^8^ and proposed the Clinical Frailty Scale (CFS)^8^^,^^9^ as a method of measuring frailty. The CFS has been widely used in the literature to objectively measure frailty and has contributed to a growing body of evidence, including systematic reviews and meta analyses, that CPR among patients who are frail is associated with significantly lower survival rates.^10^^,^^11^

National Institute for Health and Care Excellence (NICE) guidelines for the inpatient management of COVID-19 advised assessing the stability of other medical problems and using the CFS within an individualised assessment of frailty in patients admitted to hospital with COVID-19.^12^ Admission documentation at our hospital changed to include a CFS assessment for every patient. A study since this recommendation found that, during this period, 74.5% of admitted inpatients had a do not attempt (DNA)CPR agreement in place.^13^ Concerns have been raised that continuing this practice could deprive patients who are frail of potential therapy options.^14^

To protect healthcare workers, national resuscitation guidelines also changed, advising full personal protective equipment (PPE) be worn before chest compressions start, to reduce the risk of aerosol transmission.^15^ The PPE provided for this purpose at our centre was an FF3 mask, face shield and a full-length sleeved plastic gown. Training in ‘donning and doffing’ this equipment was provided to all staff in at risk areas. Although it has been difficult to find definite evidence that CPR generated sufficient aerosol to pose an infective risk, clinical skills laboratory testing found that donning PPE delayed starting CPR.^16^ In line with national guidelines at the time, CPR protocols at our centre changed accordingly.^15^

A meta-analysis of 10 studies including 1,179 patients found that ROSC was achieved in 32.9% of patients with COVID-19, whereas survival to 30 days was 10.1%.^17^ Similarly, data from the Swedish Registry of Resuscitation found that patients with COVID-19 had half the probability of surviving CPR compared with patients who did not have COVID-19.^18^ Despite the numerous published reports of COVID-19 and resuscitation, there is a paucity of information regarding CPR in the context of multiple health conditions or frailty.

We previously published an analysis of patients who received CPR at our centre between 2017 and 2018, which assessed the impact of frailty and multiple health conditions on survival from CPR.^7^ A linear relationship between increasing CFS and diminished survival at discharge, 30 days and 1 year, and a linear relationship between an increasing Charlson Comorbidity Index (CCI) and diminished survival at the same time points were identified.^7^ We also reported better outcomes for patients who had a cardiac arrest in the cardiology department compared with other wards in the hospital. In the current study, we repeated this analysis with patients who received CPR during the first year of the COVID-19 response, to compare the total number of cardiac arrest events before and during COVID-19, assessing the effect of new advanced life support (ALS) guidelines, the burden of medical emergencies on hospital wards and behavioural changes because of the pandemic response. We sought to repeat our assessment of the impact of frailty and multiple health conditions before and during the COVID-19 response. Comparison of rates of return of spontaneous circulation (ROSC), and survival rates at discharge, 30 days and 1 year were planned. Analysis focussed on the following subgroups: (a) all patients; (b) patients with COVID-19; (c) patients who were frail (according to CFS); (d) patients with multiple health conditions (according to CCI); and (e) inpatients on cardiology wards.

## Methods

A retrospective analysis of prospectively collected clinical and mortality data from contemporaneous patient notes and electronic patient records of all adult cardiac arrest calls was undertaken using the same methods as our previous study to allow direct comparison and combination of results.^7^ The period of study was from 21 June 2020 to 21 June 2021 at a 750-bed NHS tertiary hospital in South Wales, which includes an intensive treatment unit (ITU), general medicine and general surgery beds, and tertiary services, including cardiology, vascular surgery, nephrology and neurology. The hospital catchment area includes urban centres and rural areas. The choice of study period was influenced by the redeployment of hospital resuscitation officers and concerns that records earlier in 2020 would not be accurate. Advice on limiting treatment, admission documentation and ALS guidelines to include more comprehensive PPE changed in March 2020, before the start of this study period. Once again, patients who had an out-of-hospital cardiac arrest (OOHCA) or a cardiac arrest in the intensive care department were excluded from this study. Approval was sought and obtained from our health authority audit office. Statistical analyses were performed using non-parametric tests with JASP software version 16.0.2. Mann–Whitney U tests were performed to compare groups, and logistic regression was used to investigate correlations. Where possible, data were adjusted for age and shockable presenting rhythms.

## Results

### All patients

**Fig 1:**
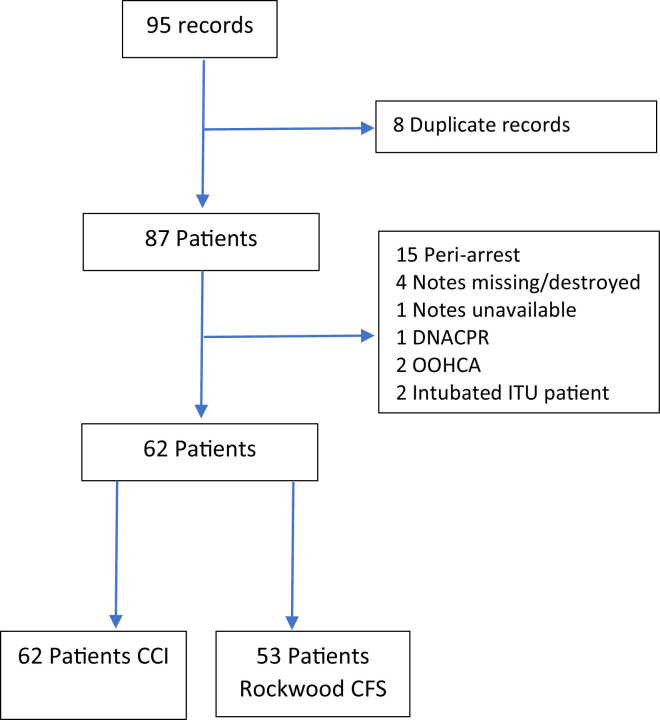
Flowchart of patients included in the study. CCI = Charlson Comorbidity Index; CFS = Clinical Frailty Scale; DNACPR = do not attempt cardiopulmonary resuscitation; ITU = intensive therapy unit; OOHCA = out-of-hospital cardiac arrest.

There was a large reduction in the total number of cardiac arrest calls made in the 2020–21 study period, with 95 alerts compared with 192 in 2017–18. After applying the exclusion criteria (Fig 1), 62 patients were included in analysis compared with 113 in the earlier study. This reduction cannot be explained by a reduction in crude admission rate during the 2020–21 study period alone (33,780/year compared with 45,430/year admissions during the 2017-8 study).

The rate of ROSC was similar among both cohorts (Table 1). Of the 62 studied patients, 20 (32.2%) survived to discharge; although this was an increase compared with a survival to discharge of 27 of 113 patients (23.7%) in 2017–18, it was not statistically significant (p=0.210). Similar rates of ROSC were observed between the two cohorts.

**Table 1:** Patient characteristics and outcomes.

	2017–18	2020–21	P value
Total admissions	45,430	33,780	–
Cardiac arrest calls	192	95	–
Patients included	113	62	–
Mean age	74	69	0.019
Median CFS	4.2	3.4	0.006
Median CCI	5.7	4.1	<0.001
Cardiology (tertiary)	44 (38.9%)	40 (64.5%)	0.002
General medicine	41 (36.3%)	21 (33.9%)	0.510
General surgery	14 (12.4%)	3 (4.8%)	0.108
Nephrology	3 (2.7%)	1 (1.6%)	0.661
ENT, T&O Vascular	11 (9.7%)	4 (6.5%)	0.461
Shockable rhythm	27 (23.8%)	22 (35.5%)	0.112
ROSC	68 (60.2%)	38 (61.3%)	0.960
Survival discharge	27 (23.8%)	20 (32.2%)	0.210
Survival 30 days	27 (23.8%)	20 (32.2%)	0.235
Survival 1 year	26 (23.0%)	19 (30.6%)	0.271

CCI = Charlson Comorbidity Index; CFS = Clinical Frailty Scale; ENT – ear, nose, throat; ROSC = return of spontaneous circulation; T&O, trauma and orthopaedics.

The median age (p=0.019), CCI (p<0.001) and CFS (p=0.006) were significantly lower in 2020–21 compared with 2017–18.

### Patients with COVID-19

Only one patient with COVID-19 received CPR on a ward in this study. Unfortunately, this person did not survive. Given the low number of patients available, the planned statistical analysis was abandoned.

### Frail (according to CFS)

#### Increasing frailty

Patients with a CFS >3 were less likely to survive 30 days (p=0.008), survive to discharge (p=0.008) or have documented ROSC (p=0.007) after CPR compared with patients who were less frail. No patient with a CFS >4 survived to discharge after CPR. A detailed breakdown of the results is provided in Table 2.

**Table 2:** Comparison of CPR outcomes in both studied populations.

	Number of patients		ROSC		30-day survival		Survival to discharge		1-year survival	
	2017–18	2020–21	2017–18	2020–21	2017–18	2020–21	2017–18	2020–21	2017–18	2020–21
Total	113	62	68 (60.1%)	38 (61.2%)	27 (23.9%)	20 (32.3%)	26 (23.0%)	20 (32.3%)	26 (23.0%)	19 (30.6%)
Clinical Frailty Scale pooled										
1–3 (not frail)	35	35	30 (85.7%)	25 (71.4%)	16 (45.7%)	14 (40.0%)	16 (45.7%)	14 (40.0%)	16 (45.7%)	14 (40.0%)
4 (pre-frail)	15	4	8 (53.3%)	1 (25.0%)	3 (20.0%)	1 (25.0%)	3 (20.0%)	1 (25.0%)	3 (20.0%)	1 (25.0%)
5–9 (frail)	39	14	21 (58%)	5 (35.7%)	5 (12.8%)	0 (0%)	5 (12.8%)	0 (0%)	4 (10.3%)	0 (0%)
Cardiology patients										
1–3 (not frail)	18	27	17 (94.4%)	19 (70.3%)	13 (72.2%)	12 (44.4%)	13 (72.2%)	12 (44.4%)	13 (72.2%)	12 (44.4%)
4 (pre-frail)	6	2	3 (50%)	0 (0%)	2 (33.3%)	0 (0%)	2 (33.3%)	0 (0%)	2 (33.3%)	0 (0%)
5–9 (frail)	6	3	6 (100%)	1 (33.3%)	3 (50%)	0 (0%)	3 (50%)	0 (0%)	3 (50%)	0 (0%)
Non-cardiology patients										
1–3 (not frail)	17	8	13 (76.5%)	5 (62.5%)	3 (17.6%)	2 (25%)	3 (17.6%)	2 (25%)	3 (17.6%)	2 (25%)
4 (pre-frail)	9	2	5 (55.6%)	1 (50%)	1 (11.1%)	1 (50%)	1 (11.1%)	1 (50%)	1 (11.1%)	0 (0%)
5–9 (frail)	33	12	15 (45.5%)	4 (33.3%)	2 (6.1%)	0 (0%)	2 (6.1%)	0 (0%)	2 (6.1%)	0 (0%)
Charlson Comorbidity Index pooled										
0	1	3	1 (100%)	3 (100%)	1 (100%)	1 (33.3%)	1 (100%)	1 (33.3%)	1 (100%)	1 (33.3%)
1–3	18	18	14 (77.8%)	13 (72.2%)	8 (44.4%)	8 (44.4%)	8 (44.4%)	8 (44.4%)	8 (44.4%)	8 (44.4%)
>4	89	41	52 (58.4%)	22 (53.7%)	18 (20.2%)	11 (26.8%)	18 (20.2%)	11 (26.8%)	17 (19.1%)	10 (24.3%)
Charlson Comorbidity Index cardiology patients										
0	1	2	1 (100%)	2 (100%)	1 (100%)	1 (50%)	1 (100%)	1 (50%)	1 (100%)	1 (50%)
1–3	7	15	7 (100%)	7 (46.7%)	7 (100%)	7 (46.7%)	7 (100%)	7 (46.7%)	7 (100%)	7 (46.7%)
>4	31	23	24 (77.4%)	15 (65.2%)	14 (45.2%)	9 (39.1%)	14 (45.2%)	9 (39.1%)	13 (41.9%)	9 (39.1%)
Charlson Comorbidity Index non-cardiology patients										
0	0	1	0	1 (100%)	0	0 (0%)	0	0 (0%)	0	0 (0%)
1–3	11	3	7(63.6%)	2 (66.7%)	1 (9.1%)	2 (66.7%)	1 (9.1%)	2 (66.7%)	1 (9.1%)	2 (66.7%)
>4	58	18	10 (17.2%)	7 (38.9%)	4 (6.9%)	2 (11.1%)	4 (6.9%)	2 (11.1%)	4 (6.9%)	1 (5.6%)

ROSC = return of spontaneous circulation.

#### Linear relationship

Logistic regression correcting for age and a shockable presenting rhythm of the 2020–21 cohort found statistically significant relationships between increasing CFS and ROSC (p=0.041), 30-day survival (p=0.010), survival at discharge (p=0.010) and 1-year survival (p=0.032).

By combining both cohorts and analysing the 175 patients together using the same logistic regression linear correlations were detected between increasing CFS and ROSC, survival at discharge, survival at 30 days and survival at 1 year (all with p<0.001).

### Multiple health conditions (according to CCI)

#### Increasing comorbidity

Compared with the rest of the population, patients with a CCI of 4 and above were not statistically less likely to achieve ROSC (p=0.057), or survive to 30 days (p=0.102), discharge (p=0.102) or 1 year (p=0.062).

#### Linear relationship

Logistic regression correcting for age and a shockable presenting rhythm of the 2020–21 cohort found statistically significant relationships between increasing CCI and reduced survival at discharge and 30 days (p=0.038 for both time points). The relationship between CFS and ROSC (p=0.088) and survival at 1 year (p=0.069) did not meet statistical significance.

However, as with CFS, after combining both cohorts and analysing all 175 patients together using the same logistic regression, a linear correlation was detected between increasing CCI and ROSC, survival at discharge, and survival at 30 days and at 1 year (all p<0.001).

### Patients on a cardiology ward

A significantly higher proportion of cardiac arrests occurred in the cardiology department in this study compared with 2017–18 (Table 2). However, the total number of patients remained similar.

Of the 40 episodes of CPR in the cardiology department, 33 (82.5%) occurred in either the catheterisation laboratory, critical care unit (CCU) or another monitored bed. In total, 17 (51.5%) patients under the care of cardiology survived to discharge after CPR. The presenting rhythm was shockable in 45.9% of all cardiology patients compared with 21.7% of other patients. A detailed breakdown of CPR outcomes for cardiology patients grouped by CCI and CFS is provided in Table 1.

## Discussion

Compared with our 2017–18 comparative study,^7^ there was an overall reduction in the number of cardiac arrest calls during the 2020–21 study period. This cannot be explained by fewer admissions to hospital alone and, thus, other influencing factors must be considered. Unfortunately, no data were available for comparison of patient demographics or clinical information for total admissions during this period.

The repeat study period occurred during a period of unprecedented global change and uncertainty resulting from the COVID-19 pandemic. In turn, greater emphasis was placed on advanced care planning.^19^ As demonstrated by our earlier study and several others,^1^, ^2^, ^3^, ^4^, ^5^, ^6^, ^7^ frailty has been demonstrated to be a good indicator of likely poor outcome from cardiac arrests^7^ and, thus, is an important factor in facilitating comprehensive advanced care plans. We also demonstrated that multiple health conditions are an important factor influencing likely survival from CPR.^7^ Observed improvement in the assessment and documentation of a patient's frailty during this period might have also been a factor triggering discussions regarding advanced care planning. As a result, overall patient selection was likely improved, such that appropriate advanced care plans for patients who were severely frail and deemed unlikely to survive cardiac arrest were placed before an arrest call was required. We believe the lack of patients with COVID-19 requiring ward-based cardiac arrest calls in the study is testimony to the hard work of the frontline teams that either placed appropriate ceilings of care earlier during the admission or ensured timely patient transfer to ITU. No data were available regarding the number of patients eligible for resuscitation before transfer to ITU. We did not find clear evidence of harm caused by the change in resuscitation guidelines in this small study, given that the rates of survival and ROSC were statistically similar between the cohorts.

The linear relationship between increasing frailty and worsening outcome from CPR previously demonstrated in our earlier study was repeated in this current research. Our findings align with others in the field, which have also demonstrated that a high CFS score is associated with poor outcome.^1^, ^2^, ^3^, ^4^, ^5^, ^6^, ^7^ However, comorbidity as a marker of poor outcome from CPR is not as well studied and this study is uniquely placed to combine the data from both studies. With our combined research data, we have shown that there is also a linear relationship between increasing multiple health conditions and worsening outcome from CPR. Individually, however, we were unable to demonstrate this definitive linear trend in our most recent research. We postulate that this is likely a result of an increasing proportion of the population being under the care of the cardiology team during their arrest. This division of care would suggest an underlying cardiological cause for their arrest and, thus, a better likelihood of success from cardiac arrest, even in those with multiple health conditions.^20^ We postulate that the combination of a higher likelihood of a reversible cause and cardiac monitoring (and, thus, more rapid response) both contribute to the improved survival trend demonstrated in this and our previous study when a patient is cared for primarily by tertiary-centre cardiologists, especially in a tertiary centre, such as used in this study. The smaller sample size in the follow-up study might have also influenced this result. Given insufficient documentation, we were unable to determine the exact number of patients not under the care of the cardiology team who received cardiac monitoring before their arrest.

Despite the novel findings of this study, we recognise that the relatively small sample size and the retrospective nature of the study design are limiting. For example, some data were lost because of incomplete documentation. Nevertheless, improved documentation in the follow-up study led to a notable comparative improvement. Ultimately, we hope that our data will contribute to a meta-analysis or encourage the formation of a data registry so that data related to changes in practice can be recorded.

## Conclusion

We were unable to find evidence that the change in ALS guidance or the increased emphasis on advanced care planning during the coronavirus response resulted in harm to patients. It is likely that the trend toward an increase in survival rate in this study was the result of fewer resuscitation attempts in patients who were frail and/or had multiple health conditions. By combining both datasets, we demonstrated that there is a linear relationship between increased CFS and reduced survival from CPR, as is the relationship between increasing CCI and survival.

**Information box**

**What is known?**

• Increasing frailty and comorbidity are both associated with worse outcome from CPR.
• ALS algorithms changed during the COVID-19 pandemic to delay chest compressions until appropriate PPE was worn, because of the possibility of aerosol generation during CPR.
• Timely advanced care discussions and DNACPR discussions were also recommended as part of the pandemic response.

**What is the question?**

• Did COVID-19-induced changes affect CPR outcomes?
• Is there further evidence that multiple health conditions are associated with worse outcomes after cardiac arrest?

**What was found?**

• There were fewer overall cardiac arrest calls compared with our previous study over a similar period.
• Patients were younger, less frail and with fewer health conditions compared with the previous study population.
• Survival to discharge increased but the change was not statistically significant.

**What is the implication for practice now?**

• We hope these additional data will provide confidence to those initiating DNACPR discussions with patients who are frail and/or have multiple health conditions.
• Given that outcomes were no worse during the COVID-19 pandemic, we did not find evidence of harm caused by the change in ALS guidelines.

Alt-text: Unlabelled box
